# Advances in 3D Printed Electronics: Materials, Processes, Properties and Applications

**DOI:** 10.3390/ma16175975

**Published:** 2023-08-31

**Authors:** Haibin Tang, Xingzhi Xiao

**Affiliations:** 1 School of Intelligent Manufacturing, Nanjing University of Science and Technology, Nanjing 210094, China; 2 School of Mechanical Engineering, Nanjing University of Science and Technology, Nanjing 210094, China; xingzhi.xiao@njust.edu.cn

The current Special Issue entitled “Advances in 3D printed electronics: materials, processes, properties and applications” aims to discuss the latest developments in the field of the AM of structures or components with reinforcements [1] or conductive elements [2].

Additive manufacturing (AM) shows prominent advantages in the modern industry owing to the almost unlimited design freedom it provides to produce components with complex 3D shapes without the need for tooling [3,4]. In order to fabricate structures or components for real-world applications with high performances or multiple functions, reinforcements or conductive elements are introduced in the latest AM techniques. The methodology and mechanism of multi-functional AM are meaningful to its further development.

The topics addressed in this Special Issue include new computational models and approaches to predicting the fabrication processes and mechanical properties of complex structures and components and new additive manufacturing technologies that cover various types of material extrusion, material lamination, binder jetting, ink writing [5], selective laser sintering [6], curing and sintering, etc. New composite systems containing either fiber reinforcements or conductive elements are covered. The applications of the AM of integrated structures in other fields, e.g., the design of robots and bio-inspired structures, are also welcome.

## References

[1] Tang H., Sun Q., Li Z., Su X., Yan W. (2021). Longitudinal compression failure of 3D printed continuous carbon fiber reinforced composites: An experimental and computational study. Compos. Part A Appl. Sci. Manuf..

[2] MacDonald E., Wicker R. (2016). Multiprocess 3D printing for increasing component functionality. Science.

[3] Tang H., Huang H., Liu C., Liu Z., Yan W. (2021). Multi-scale modelling of structure-property relationship in additively manufactured metallic materials. Int. J. Mech. Sci..

[4] Tang D., Yang K., Gao T., Liu T., Tang H. (2023). Mechanical-electromagnetic integration design of Al_2_O_3_/SiO_2_ ceramic cellular materials fabricated by digital light processing. Thin-Walled Struct..

[5] Tang D., Tang H. (2022). Self-healing diamond/geopolymer composites fabricated by extrusion-based additive manufacturing. Addit. Manuf..

[6] Tang H., Chen H., Sun Q., Chen Z., Yan W. (2021). Experimental and computational analysis of structure-property relationship in carbon fiber reinforced polymer composites fabricated by selective laser sintering. Compos. Part B Eng..

